# Evaluation of Biomarkers of Bone Metabolism on Salivary Matrix in the Remodeling of Periodontal Tissue during Orthodontic Treatment

**DOI:** 10.3390/dj12070209

**Published:** 2024-07-09

**Authors:** Angela Pia Cazzolla, Vincenzo Brescia, Roberto Lovero, Antonietta Fontana, Arcangela Giustino, Mario Dioguardi, Maria Severa Di Comite, Francesca Di Serio, Domenico Ciavarella, Vito Crincoli

**Affiliations:** 1 Department of Clinical and Experimental Medicine, Università degli Studi di Foggia, 71100 Foggia, Italy; mario.dioguardi@unifg.it (M.D.); domenico.ciavarella@unifg.it (D.C.); 2 Clinical Pathology Unit, AOU Policlinico Consorziale di Bari—Ospedale Giovanni XXIII, 70124 Bari, Italy; bresciavincenzo58@gmail.com (V.B.); rlovero@tiscali.it (R.L.); a-fontana@libero.it (A.F.); francesca.diserio@policlinico.ba.it (F.D.S.); 3 Department of Biomedical Sciences and Human Oncology, Aldo Moro, University of Bari, 70121 Bari, Italy; arcangela.giustino@uniba.it; 4 Department of Basic Medical Sciences, Neurosciences and Sensory Organs, Human Anatomy Section, Aldo Moro, University of Bari, 70121 Bari, Italy; mariasevera.dicomite@uniba.it; 5 Interdisciplinary Department of Medicine, Aldo Moro, University of Bari, 70121 Bari, Italy

**Keywords:** PINP, PTHrP, TRAcP, bone biomarkers, orthodontic treatment critical difference (RCV)

## Abstract

The aim of this study was to evaluate changes in the concentration of N-terminal type I collagen extension pro-peptide (PINP), tartrate-resistant acid phosphatase (TRAcP), and parathyroid hormone-related protein (PTHrP) in saliva during orthodontic treatment in order to evaluate whether changes in bone turnover marker (BTM) concentration can help highlight the effects of orthodontic mechanical loading in the absence of clinical evidence of tooth movement in terms of tooth movement. Saliva samples from 25 apparently healthy young subjects (10 females and 15 males) were collected using Salivette^®^ (Sarstedt) with cotton swabs and the concentrations of PTHrP, TRAcP 5b, and PINP were analyzed at time 0 (T1), 25 days (T2), and at 45 days (T3). Differences in the median value of biomarker levels between baseline T1 and follow-up of the different groups (T2 and T3) were assessed using the non-parametric Mann–Whitney U test. Trough concentrations of P1NP, PTHrP, and TRAcP were 0.80 µg/L, 0.21 ng/mL, and 0.90 U/L above the method LOD. The non-parametric Mann–Whitney U test confirmed a statistically significant difference in T1 versus concentrations of T2 and T3. All subjects evaluated had a statistically significant difference between T1 vs. T3. when compared with the specific critical difference (RCV) for the analyte The results obtained demonstrate that the evaluation of BTM changes in saliva can help the evaluation of orthodontic procedures and the monitoring of biomechanical therapy.

## 1. Introduction

Orthodontists generally aim to achieve ideal orthognathic conditions with suitable treatment times [1]. Fixed orthodontic treatment is based on the application of a system of mechanical tension and pressure forces on the teeth and the supporting tissues of the tooth (periodontal ligament and alveolar bone) [2,3].

The application of these forces induces a cellular response on the periodontal ligament (PDL), which also has an effect on the alveolar bone, with different remodeling phenomena [4,5,6,7].

The mechanical stress induced by fixed orthodontic forces activates a cascade of cellular responses with an inflammatory process affecting the periodontal ligament (PDL) and the alveolar process with consequent bone remodeling [8].

Mechanical forces modify the vascular microenvironment and determine the local release of inflammatory mediators such as interleukin 1b (IL-1β), involved in bone remodeling, and tumor necrosis factor alpha (TNF-α), involved in bone resorption [9,10,11,12].

The bone remodeling process also involves interleukin-8 (IL-8) in tension sites and the prostaglandins PGE1 and PGE2 in the PDL resorption sites [13]. In fact, several studies show significant concentrations of inflammatory markers in the saliva and crevicular fluid of subjects undergoing orthodontic therapies, although it is not entirely clear whether the presence of these cytokines is linked to the action of orthodontic forces or to the periodontal aseptic inflammatory process with the release of cytokine [4,12,14,15,16,17,18,19,20,21].

The timing of the phases of fixed orthodontic movement has been studied by several authors [22,23] including Pilon et al. who identified four phases. The initial phase occurs approximately within the first 2 days after the application of force and is characterized by rapid tooth movement within the alveolus. The arrest phase lasts 20–30 days, during which the tooth is immobile in a phase of stasis to reach the maximum extension limit of the periodontal fibers, and ends when the osteoclasts of the endosteal wall are activated and reabsorb the hyaline substance (indirect resorption). The phase of acceleration or secondary displacement lasts 40 days and is characterized by a continuous tooth movement in the direction of the reabsorption area. The linear phase is characterized by overall tooth movement, important cellular recruitment (macrophages, fibroblasts, osteoblasts, and osteoclasts), and an increase in the activity of biomarkers of bone metabolism [24,25,26].

Biomarkers are biologically active substances responsible for a complex network of cell–cell and cell–matrix interactions, which can be measured and evaluated objectively [5]. These biomarkers reflect all these phases of orthodontic tooth movement and can be found in the gingival crevicular fluid (GCF) of moving teeth with significant elevations in the concentrations of saliva [5,27,28,29,30].

Although three-dimensional intraoral imaging has aroused great interest in dentistry as a means for interpreting the results of orthodontic therapy [31], it is currently believed that biomarkers, can be objectively measured and evaluated for the same purposes. These biomarkers reflect all phases of orthodontic tooth movement and can be found in gingival crevicular fluid (GCF) and saliva with significant increases in concentrations [5,32,33,34].

The evaluation of the concentrations of bone remodeling biomarkers in saliva, being a practical and simple procedure, is suitable to provide indications of the biological phenomena taking place and to obtain information on the quantity and duration of the force to be applied during orthodontic tooth movement (OTM) [5,35].

Saliva, has proven to be particularly useful in children due to the non-invasive nature of sampling and the ease of collecting the fluid. However, the concentrations of biomarkers in saliva can be influenced by the presence of blood in the oral cavity, by use of drugs, or by the presence of systemic pathologies capable of modifying or inhibiting the function of the salivary glands [36,37,38,39,40,41,42,43].

Some bone turnover markers (BTMs) represent products of bone proteins, particularly type I collagen which undergoes substantial post-translational modifications during the synthesis of new bone [44]. Other BTMs are products of bone cells and reflect the activity and number of osteoblasts or osteoclasts within the bone environment at a given time [45].

Different guidelines recommend using a serum marker of bone formation integrated with a marker of resorption for the evaluation of bone turnover, selected based on the clinical context, performance in clinical studies, wide use, and relatively low analytical variability [46,47,48,49,50].

All biomarkers that meet these requirements can be used to describe the biological changes that occur during OTM [26], with a single limitation determined by the need that the concentrations of the analyte present in the saliva are not higher than the limit of detection (LoD) of the selected method.

Procollagen I, is synthesized by osteoblasts, and the terminal propeptides of the molecule are cleaved off extracellularly. The circulating concentration of the N-terminal propeptide of procollagen I (PINP) is correlated to that of osteoblastic activity, which is normally coupled to osteoclastic activity in the bone resorption phase [51].

Its levels are equimolar to those of collagen incorporated into the bone matrix and correlate significantly with histomorphometry and bone formation measures. Its dosage in saliva could be useful, as it is a marker of bone formation and reflects the activity of osteoblasts [52].

Another biomarker that offers notable insights into studies involving saliva is TRAcP, a 35–37 kDa glycoprotein and isoenzyme of acid phosphatase (ACP). It is produced by osteoclasts [53] and released into the bone resorption gaps. It helps the migration of osteoclasts and plays a role in degrading type I collagen in the bone matrix [54,55]. TRACP-5b concentration is known to reflect bone resorption; however, it must be noted that it reflects osteoclast number rather than osteoclast activity [56,57].

PTHrP carriers out autocrine and paracrine hormonal activities, such as the regulation of bone development. [58].

It is expressed only in the cells of the normal dental pulp and carries out a particularly evident regulatory activity during the maturation of the teeth when it intervenes to regulate the spatial coordination of the bone cellular activity. In fact, it acts on the osteoclasts for the necessary resorption processes of the bone overlying the crown, and on osteoblasts to form the bone at the base of the tooth and push it toward the top of the crypt [59,60,61,62,63].

It is clear that several biomarkers can describe the biological changes that occur during bone remodeling following orthodontic treatment. However, they are not widely implemented in clinical practice [26].

The aim of this study was to investigate, in the initial phases of orthodontic treatment, the variations in the salivary concentration of some BTMs measured with a sensitive and specific immunometric method, in order to evaluate the effects of mechanical loading in the absence of clinical evidence of tooth movement.

## 2. Materials and Methods

The study was conducted at the Foggia Dental Clinic from September 2021 to February 2023 in collaboration with the Clinical Pathology Unit of the Bari Polyclinic. A total of 25 patients were recruited, all of whom presented with class I dental and skeletal relationships, good gingival and periodontal health, aged between 15 and 18 years, and with upper and lower dental crowding assessed between 2.1 and 4.0 mm [64,65]. The study was conducted in accordance with the Helsinki Declaration and approved by the Ethical Committees of the Policlinico University Hospital of Bari (Biomarkers of Bone Metabolism, Study number. 38359/COMET of 27 April 2021 BMOPed) [66].

### 2.1. General Exclusion Criteria

Individuals with liver and/or kidney diseases, inflammatory bowel diseases, diabetes mellitus, and cardiovascular diseases/disorders were excluded.

Patients who had received immunosuppressive drugs in the last three months, pregnant or lactating females were also excluded. Smoking subjects, subjects who had carried out extreme physical activity in the previous days subjects presenting bleeding buccal lesions of any nature, including mechanical ones, during each sample collection phase were excluded.

#### 2.1.1. Periodontal Exclusion Criteria

Patients who presented with Bleeding on Probing (BOP) greater than >10%, Probing Pocket Depth (PPD) greater than 3 mm, Loes and Sillness Gingival Index (GI) greater than 1, or Full Month Plaque Score (FMPS) greater than 20% were excluded. Furthermore, all patients who had undergone orthodontic or periodontal treatments in the last few months, who had piercings in the tongue or lower anterior lip, and who had not undergone regular and periodic dental check-ups were excluded. The definition of periodontitis followed the 2017 classification of periodontal diseases and conditions [66] and the Silness and Loe Plaque Index (PI), GI, BOP, PPD, and FMPS were assessed at each visit.

Index evaluation was performed on the control molar and on six sites for each tooth.

From 1 week to 1 month before salivary collection, participants underwent a professional supra- and subgingival scaling session and also received repeated oral hygiene instructions.

Clinical parameters (BOP, PPD, GI, FMPS, and PI) were measured for all existing dentition, except wisdom teeth which were excluded from the evaluation.

All included subjects were advised to maintain good oral hygiene during orthodontic treatment. Participants’ oral hygiene levels were periodically assessed through their GI scores. A GI score of less than 1 is considered a good indicator of oral health.

#### 2.1.2. Orthodontic Evaluation

The orthodontic treatment involved direct bracket bonding from UR7 to UL7 and from LL7 to LR7 according to the Roth technique, using vestibular fixed brackets with a 0.022 slot (In-Ovation R brackets 0.022″ slot Dentsply GAC International, The Hague, Netherlands) and light-curing resin composite Transbond ™ (3M Unitek, Monrovia, CA, USA). Photo-polymerization was performed for 20 s by using a high-power led lamp (Elipar S10™, 3M ESPE). Intraoral scans of the upper and lower dental arches were acquired using an intraoral scanner (Carestream Dental CS3600, Atlanta, GA, USA) before bonding and after 45 days (with brackets and orthodontic arch inserted) to evaluate the resolution of crowding during the three phases of the study. An initial arch wire of 0.014″ Sentalloy 80 gr (Dentsply GAC, Islandia, New York, NY, USA) was applied and used for alignment for the entire duration of the study (45 days) and never replaced during all three phases of the study. A total of 25 recruited patients presented class I dental and skeletal relationships and upper and lower dental crowding (TSALD tooth-size/arch length discrepancy) evaluated between 2.1 and 4.0 mm [64,65].

All patients who had shown an improvement in crowding greater than 0.5 mm were excluded as 0.5 mm represents the limit of accuracy and precision of the scanner used [66,67,68].

For the superimposition of the scans and measurement of the digital models, 3D-Slicer software (version 5.0.2) was used. STL files have been imported and each model was aligned parallel in all directions.

### 2.2. Salivary Sample Collection

The salivary samples were collected 3 times: before the start of orthodontic treatment (T1), 25 days after the start of orthodontic treatment (T2), and 45 days after the start of treatment (T3) [24].

All samples were collected under the strict supervision of healthcare personnel to verify the suitability of the sampling phase.

To avoid errors in the salivary sample collection phase, all subjects were provided with written recommendations for the preparatory phase.

All subjects were required to fast for 2 h before saliva collection (excluding water) and to clean the mouth with water rinses without disinfectants and/or mouthwashes.

The collection of salivary samples was performed after a specialist oral examination with the passive salivation method [69]. For this purpose, Salivette^®^ (Sarstedt, Numbrecht, Germany) was used for the hygienic collection of total saliva. The collection of saliva with the Salivette^®^ method involves the use of a sterile synthetic fiber cotton roll. The execution procedure involved delicately chewing the swab for two minutes and transferring the swab into the appropriate container without manipulation. At the end of the execution and saliva collection phase, all subjects who presented saliva samples with evidence of contamination with blood were excluded from the study. Suitable samples were stored at a temperature between 4 and 8 °C until delivery to the laboratory within two hours of collection.

### 2.3. Verification of Blood Contamination on the Salivary Samples

The concentration of bone metabolism biomarkers in blood is higher than in saliva. Therefore, contamination of saliva with blood, even if not macroscopically evident, can influence their dosage. For this reason, all samples were evaluated by an automated spectrophotometric method (HIL) on a Dimension VISTA 1500 clinical chemistry analyzer (Siemens Munich, Germany) (LoD Hgb: 50 mg/dL) and all samples with Hgb were excluded.

### 2.4. Analysis of Bone Metabolic Biomarkers (BMTs)

The samples found suitable were stored at 4–8 °C for 24 h, centrifuged at 4000× *g* for 3 min, and stored at −30 °C until analysis.

The study involved the dosage on a salivary matrix of Parathyroid Hormone-Related Peptide (1–64) (PTHrP) Procollagen type I N propeptide (PINP), Tartrate Resistant Acid Phosphatase isoform 5b (TRAP5b). Parathyroid hormone-related protein (1–64) (PTHrP) assay was measured with the “competitive” enzyme immunoassay (ELISA) designed to measure the (1–34) subunit (Parathyroid hormone-related protein) (PTHrP) (1–34) EIA Kit, (Catalog No. EK-056-04) (PHOENIX PHARMACEUTICALS, INC.; 330 Beach Rd. Burlingame, CA 94010, USA) (measuring range 0.033–6000 ng/mL, Limit of Detection (LoD) 0.033 ng/mL, Analytical Coefficient of Variation (CVA) 9%). The test involved the use of the DSX ^®^ TGSTA Dynex Technologies, Inc. TRAcP isoform 5b (IDS-iSYS TRAcP 5b) (BoneTRAP^®^) (Catalog No. IS-4100) (Immunodiagnostic Systems Ltd. 10 Didcot Way, Boldon Business Park, Boldon, Tyne and Wear, NE35 9PD, UK), with a Limit of Detection (LoD) of 0.9 U/L, a linear range of 0.9–14.0 U/L, and a Coefficient of Analytical Variation (CVA) of 4.5%. PINP (IDS-iSYS Intact PINP) (Catalogue No. IS-4000) (Immunodiagnostic Systems Ltd. 10 Didct Way, Boldon Business Park, Boldon, Tyne and Wear, NE35 9PD, UK) with a Limit of Detection (LoD) of 2–230 ng/mL, a linear range of 2–230 ng/mL, and an Analytical Coefficient of Variation (CVA) of 5.2% was performed with chemiluminescence assay using TGSTA Technogenetics instrumentation (Technogenetics, Milan, Italy).

All tests were performed in accordance with the manufacturer’s instructions regarding procedures for establishing and verifying analytical quality objectives. To this end, multilevel internal quality control (IQC) materials provided by the manufacturing company and the results of External Quality Assurance (EQA) programs were used to verify the repeatability. The CVA obtained falls perfectly within the indicated limits and the EQA verification did not provide evidence of out-of-control analyses [69,70].

### 2.5. Statistic Analysis

Verification of the suitability of the sample size was carried out through the statistical power study. A significance > 95% was considered suitable [71].

The descriptive statistics of the concentrations of bone metabolism biomarkers obtained in the saliva of 25 subjects reported means, medians, distribution at 95% Confidence Interval (CI) and range, and stratification of the results at times T1, T2, and T3. The D’Agostino–Pearson test was used to evaluate the normality of the distribution of the results. A *p* value < 0.05 was considered statistically significant. Boxplots of the distribution of BMT value were used to verify the presence of any outside events to be excluded from the statistical evaluation.

The nonparametric Mann–Whitney U test was used to compare the significance of the difference between the medians of individual salivary biomarkers stratified by sampling time (T1, T2, and T3). A *p* value < 0.05 was considered statistically significant.

Multiple Comparison Graphs reporting the individual determinations and a connecting line for the median were used to evaluate the trend of the distribution of the concentration of BMT cells with respect to sampling time.

The percentage change in the concentration of the biomarkers measured at T1, T2, and T3 in the individual subjects was evaluated.

The critical difference (RCV) of the biomarkers on salivary matrix (21.18%, 56.81%, 68.44% respectively for P1NP, TRAcP and PTHrP)^70^ was used to evaluate the significance of the variation obtained [70].

The multiple variable grap.hs were used to visualize the different concentrations of the biomarkers at T1, T2, and T3 for each individual included in the study.

For statistical analysis, the MedCalc software program, version 11.6.1.0 (MedCalc Software, Mariakerke, Belgium) and Analite.it were used.

## 3. Results

### Subject Clinic

A total of 25 young subjects were enrolled in the present study. Their average age was 15.6 years, and 10 subjects were female. The power analysis of the sample size provided for 25 patients with a significance of 95. Power analysis using G*Power software (ver. 3.1.9.7; Heinrich-Heine-Universität Düsseldorf, Düsseldorf, Germany) indicated that the required minimum sample size was 25 subjects to determine this effect size with 95% power and a significance level of 5% [71]. For this calculation, the values of the mean, median, and standard deviation of PINP, TRAcP, and PTHrP at the different sampling times were taken into consideration.

The clinical gingival condition was evaluated at the beginning and during the experimental period. All patients maintained good oral hygiene throughout the study. No significant changes in the PI, BOP, PPD, and FMPS indexes were found. At each orthodontic check-up, all patients had GI < 1. Salivary biomarker values were tested 24 h after insertion and were used for comparison with subsequent measurements.

The descriptive statistics of the distribution of the concentrations of the different biomarkers, the distribution at 95% Confidence Interval (CI), and the evaluation of the normal distribution (D’Agostino–Pearson test) are reported in Table 1.

The evaluation with the D’Agostino–Pearson test showed a parametric distribution of the data. Minimum concentrations of PINP, PTHrP, and TRAcP were 0.80 µg/L, 0.21 ng/mL, and 0.90 U/L above the LoD of the method. The distribution range was 8.04 µg/L, 2.73 ng/mL, and 6.03 U/L for PINP, PTHrP, and TRAcP, respectively.

The descriptive statistics of the distribution of the concentrations of the different biomarkers stratified as a function of the times of sampling (T1, T2, and T3), including the distribution at the 95% Confidence Interval (CI), and the evaluation of the normal distribution (D’Agostino–Pearson test), are shown in Table 2.

The data obtained highlighted a progressive increase in the median concentrations from T1 to T2 and T3 in all the biomarkers evaluated, although with different trends.

Median concentrations T1 vs. T3 ranged from 2.10 to 5.20 µg/L for PINP, from 0.21 to 0.52 ng/mL for PTHrP, and from 0.90 to 2.01 U/L for TRAcP.

The D’Agostino–Pearson test rejects normality in T1 and T3 for PINP, in T2 for PTHrP, and in T2 and T3 for TRAcP.

**Figure 1:**
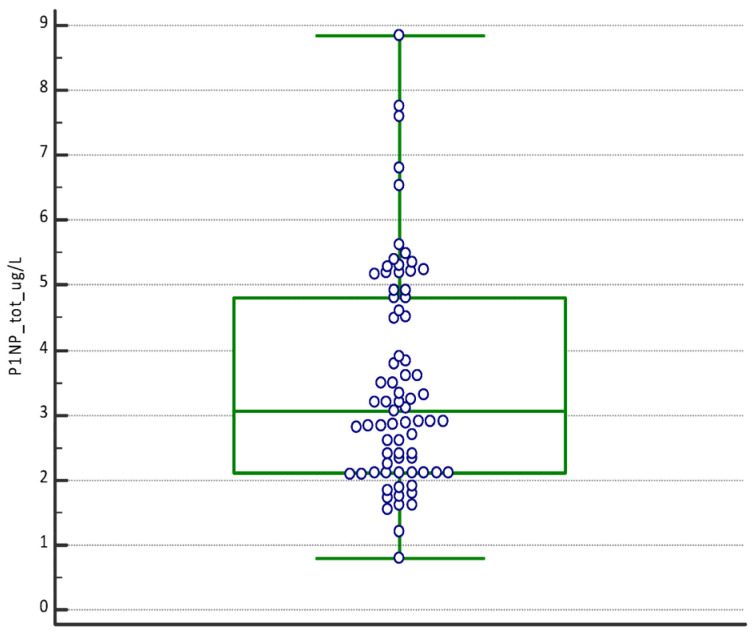

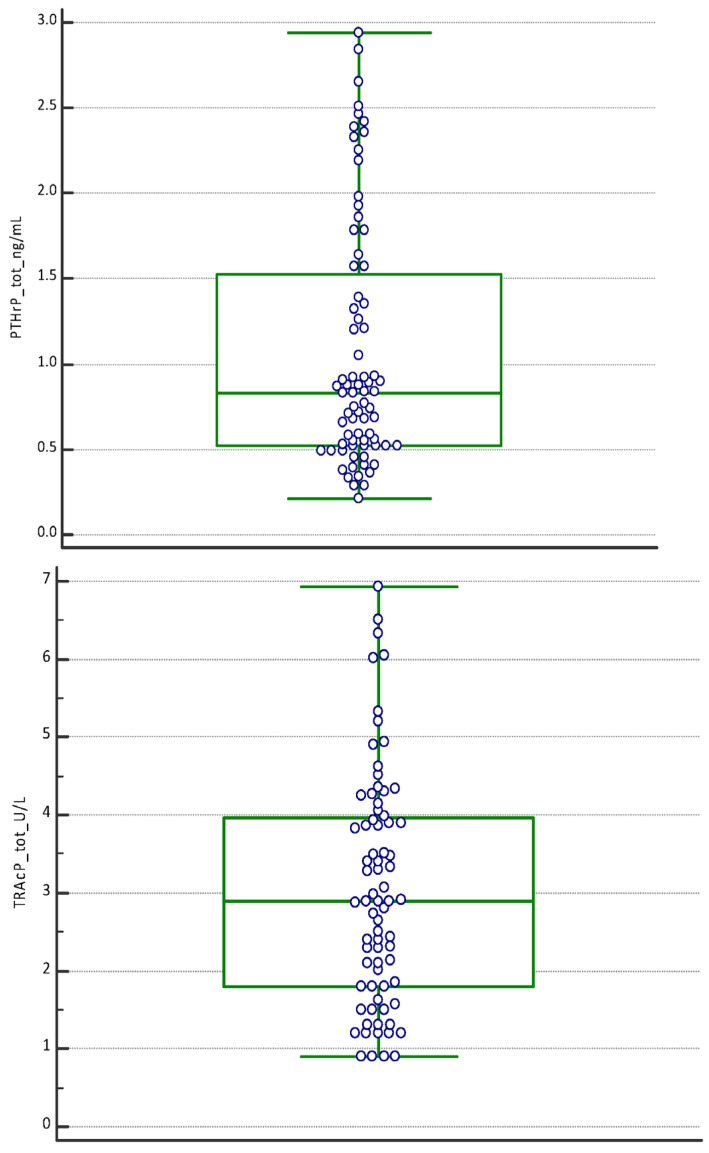
Boxplot with the statistical summary of the concentration of PINP (μg/L), PTHrP (ng/mL), and TRAcP U/L. The boxplots report the values from the 25th to the 75th quartile, the central line as the median, and the horizontal lines as the extension from the minimum to the maximum value (range) of the concentrations.

The boxplot of the PINP tot (μg/L), PTHrP tot (ng/mL), and TRAcP tot (U/L) confirms the absence of any outside values included (Figure 1).

The non-parametric Mann–Whitney U test, used to compare the significance of the difference between the median concentrations of salivary biomarkers stratified by sampling period (T1, T2, and T3), highlighted statistically significant differences by sampling period (T1, T2, and T3) (Table 3).

**Figure 2:**
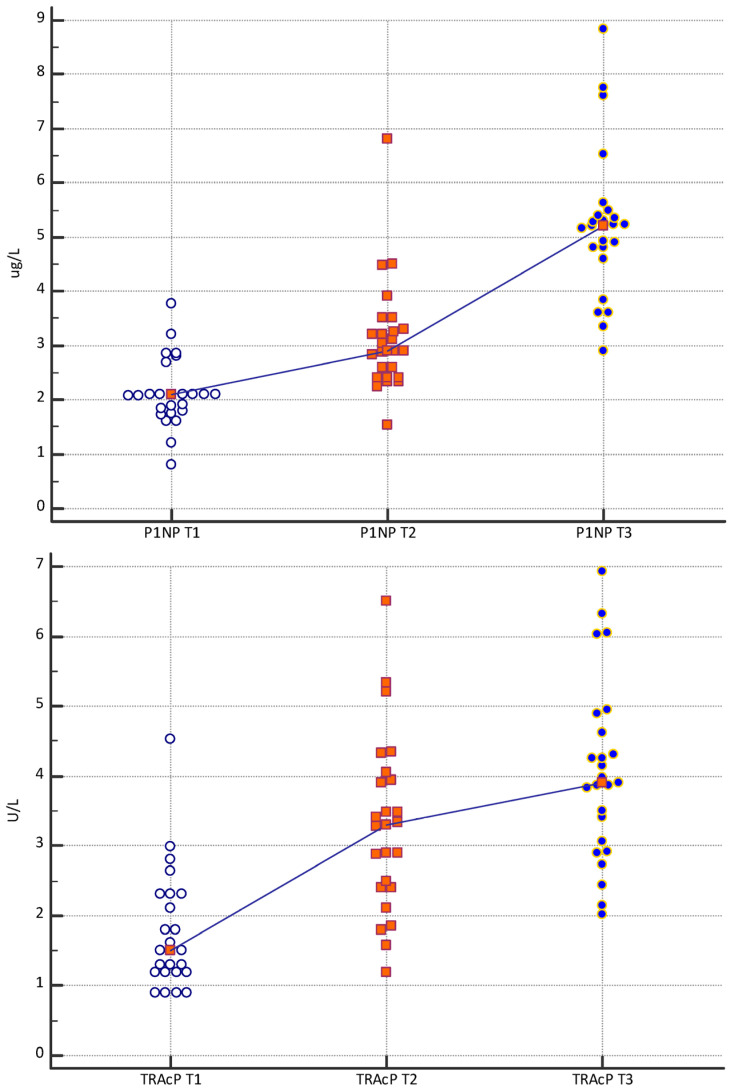

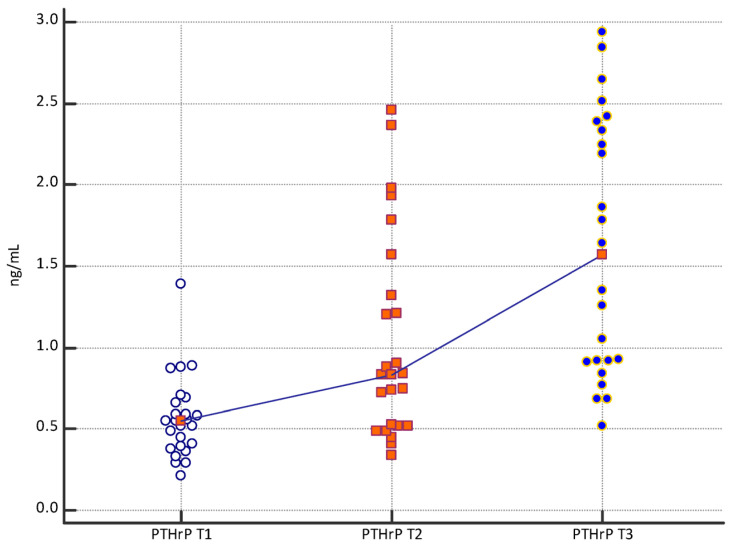
Multiple Comparison Graphs of the concentrations of PINP (μg/L), PTHrP (ng/mL), and TRAcP (U/L) in subjects evaluated and stratified at time T1, T2, and T3. The figures represent the dots, the ranges, the median values, and the trend line.

The “Multiple Comparison Graphs” relating to the bone biomarkers PINP, TRAcP, and PTHrP showing the distribution of the analyte concentration measurements with respect to the time of sampling are shown in Figure 2. The trend line connects the median of the values to the different relative times. Each biomarker shows a progressive increase from T1 to T3.

The percentage change in biomarker concentrations measured at T1, T2, and T3 is reported in Table 4.

The significance of the change was assessed using the RCV of the single marker.

The change in T2 versus T1 concentrations was significant in 19 (76%) subjects for PINP, in 18 subjects (72%) for TRAcP, and in 11 subjects (44%) for PTHrP.

All subjects evaluated had a statistically significant difference between T2 vs. T3.

**Figure 3:**
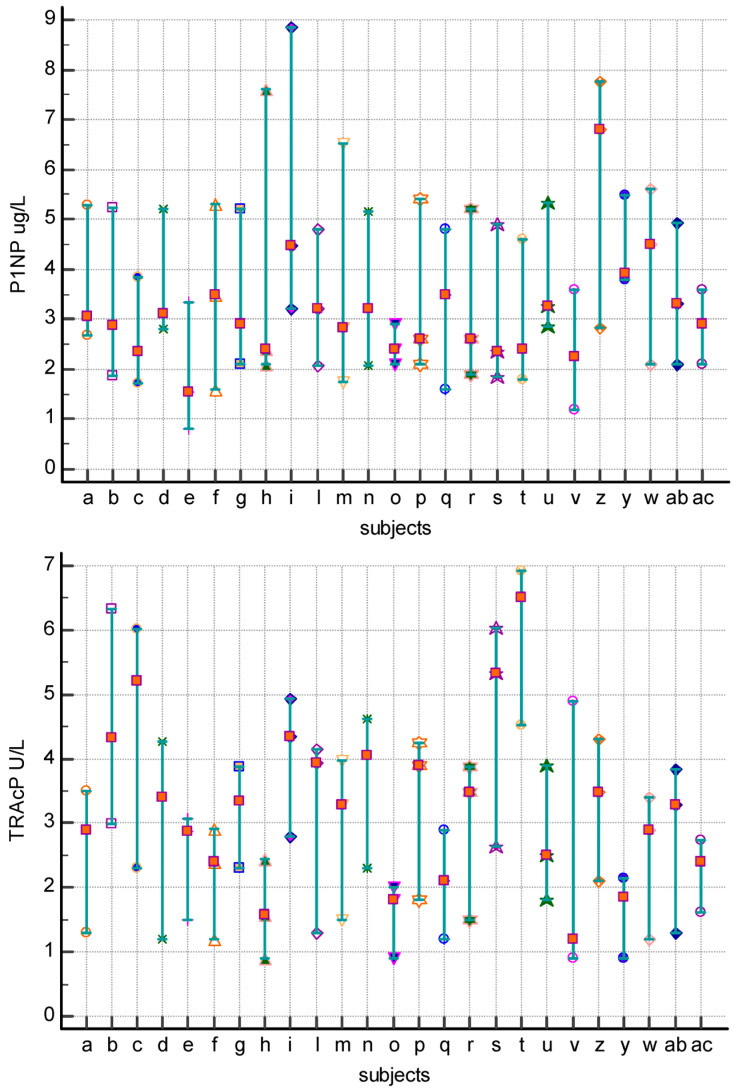

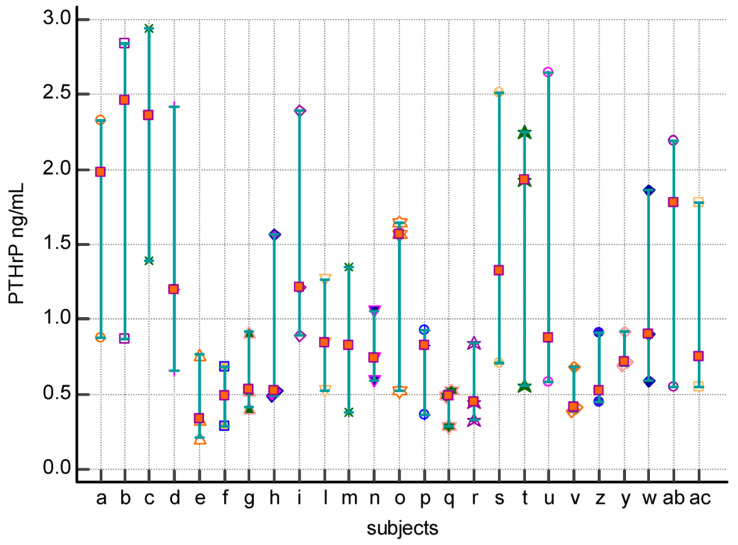
Multiple Comparison Graphs differentiated by individual analyte PINP (**a**), TRAcP (**b**), and PTHrP (**c**). The values of the biomarker obtained in the individual subjects have been reported. Red, light blue, and blue dots for T1, T2, and T3, respectively.

The “Multiple Comparison Graphs” showed the distribution of the values obtained in the individual subjects (a–ac) included in the study as a function of time (Figure 3).

The Multiple Comparison Graphs highlighted the high individuality of the concentration of biomarkers in the individual subject in fact concentrations did not cover the entire range of distribution of values.

## 4. Discussion

The periodontal ligament, the alveolar bone, and the gum are involved in a series of histological and biochemical reactions that are generated by and related to OTM determined by orthodontic therapies [4,72,73,74,75].

The application of a controlled mechanical force on the teeth activates a cascade of events in the alveolar and periodontal bone that allows tooth movement [76].

The mechanical stimulus of the OTM causes inflammatory responses in periodontal tissues and alterations in blood flow, as well as the formation and release of various biochemical mediators [4,75].

The force produced by orthodontic systems results in greater resorption on the pressure side and greater bone formation on the tension side [4,74,75,77,78].

The release of BTM into saliva is consequent to the state of remodeling of the bone as a whole; the physiology of the basic multicellular units of the bone begins with the resorption phase by osteoclasts which have a short lifespan compared to osteoblasts (2 weeks versus 3 months) [79].

Consistently in this study, TRAcP changed more rapidly after orthodontic treatment, while PINP changed in its concentration later as a consequence of subsequent new bone formation. The apparent absence of a regular and specific variation of PTHrP over time and a highly individual trend supports the hypothesis of a paracrine secretion of the hormone aimed at a local regulatory activity rather than a direct action on the bone [5,70,80].

The variation in biomarker concentrations over time suggests that the process may be an exaggerated result of physiological turnover following mechanical stimulation [81].

Other authors have evaluated the concentrations of biomarkers in the saliva of patients undergoing fixed orthodontic treatment before and 14 days after the initial application of orthodontic forces, but they did not obtain significant variations between serial determinations [3].

This study involved the collection of three salivary samples: before the start of orthodontic treatment (T1), 25 days (T2), and 45 days after starting treatment (T3). The results suggested that it is possible to have information on the state of bone remodeling in the individual subject, especially if monitoring is carried out for a sufficient time to allow the activation of the remodeling processes; 45 days after the application of an orthodontic appliance was found to be a suitable time.

To reduce the potential clinical and methodological heterogeneity of the study, data were provided on the relationships between dosages and physiological or pre-analytical factors that would have contributed to the variability of BTM concentrations. The inclusion criteria involved the selection of subjects in good general and periodontal health without a history of orthodontic and periodontal therapy during the months before the saliva collection. None of the samples were collected within 30 min of consuming a meal or drink, all subjects avoided smoking and brushed their teeth after and not before collecting saliva [13,27]. The decision to use the assay of analytes capable of expressing the results obtained in standardized units, the use of objective analytical quality values for these markers, and the use of measurement with monoclonal antibodies made it possible to obtain comparable measurements [42,43].

To allow unambiguous interpretation, it was necessary to express the data in quantitative mode according to specific detection limits and within the analytical range of the method [82,83,84].

For PINP, TRAcP, and PTHrP, the detection/quantification limits have been assessed, as these are essential analytical elements for the interpretation of the results. Biomarkers that are not detected are not necessarily absent, but concentrations are likely to be below or near the limit of detection with the analytical methods used.

Contamination can lead to false positive detection of biomarkers. In this study measures have been taken to avoid contamination of saliva with blood; a preliminary clinical and laboratory evaluation has been performed to exclude samples with potential contamination [69,76,85].

The saliva collection method involved the use of the Salivette device (Sarstedt, Numbrecht, Germany) used for the dosage of other salivary proteins [86].

Subjects were advised to chew the absorbent cotton for 2–3 min; subsequently, the samples were stored at 4–8 °C. The simplicity of salivary collection and sufficient standardization suggested that periodic measurement of BMT on saliva may represent a simple and effective way to early identify insufficient responses to orthodontic treatment even in pediatric subjects.

It was considered that the evaluation of the dynamic process of bone remodeling during OTM does not lend itself to being interpreted on the measurement of a single bone biomarker. Therefore, the variation in monitoring a biomarker of bone deposition (PINP) and resorption (TRAcP) was more useful for detecting the dynamics of the metabolic imbalance [87].

The use of BTMs for treatment monitoring requires a baseline evaluation with repeated measurement at a defined time during orthodontic treatment. To do this effectively, it is important to monitor the effect of the treatment in the individual, having as a yardstick for evaluating the clinical effectiveness the possibility of evaluating the results in the individual subject on the basis of pre-established interpretative diagnostic criteria [70]. A change in the salivary level of a single bone marker, observed in a patient during OTM, can be interpreted in light of the biological variability of the respective marker [70]. The availability of data on the significant percentage deviation (RVC), determined by biological variability and analytical variability [88], limited the errors in the interpretation of the responses on the variation in concentrations and allowed an objective evaluation of the variations [70]. Based on RCV, all subjects included in the study presented a significant change in TRAcP and PINP concentrations 45 days after the application of the orthodontic appliance, despite presenting specific concentration variations for each subject (inter-individual variability). The study of changes in biomarkers linked to bone turnover can introduce new possibilities in orthodontics to understand bone growth and remodeling. Knowledge of the process that occurs in the periodontal tissues during orthodontic therapies can lead to the correct choice of mechanical load; this would allow the treatment period to be optimized and the adverse effects associated with orthodontic treatment to be avoided.

The amount and duration of force to be used during OTM could be decided based on knowledge of the levels of these biomarkers evaluated on the basis of a deviation that is greater than the critical difference. Previous studies have shown that TRAcP provides information on bone resorption [89] and shows a peak in the fifth week, at the site of compression; TRAcP levels were greater at the 150 g strength compared to the 100 g strength [76,90].

Salivary diagnostics require adequate identification and validation of biomarkers for pathological conditions. A biomarker is a parameter that can interact physiologically and biochemically at the molecular or cellular level, and acts sequentially as an indicator of normality, pathological behavior, and in response to therapy. The analytical validation of biomarkers in the salivary matrix and their clinical usefulness in the dental field are fundamental for activating initiatives and creating reliable models for diagnosis and treatment also with the help of innovative technologies such as point-of-care (PoCT) [91].

The impact of salivary diagnostics on the healthcare system is enormous, being a non-invasive, convenient method with excellent credentials, and the introduction of BMTs in the evaluation of the dynamic process of bone remodeling during OTM, evaluated as a function of the critical difference (RCV), will raise performance standards.

Currently, the methods of evaluating and monitoring orthodontic treatment are based on clinical evidence and/or the use of intraoral scanners. These methods are not sufficient to highlight very small tooth movements and do not give adequate information regarding what happens at the level of the alveolar bone and periodontal ligament during orthodontic treatment. Such information, on the contrary, could be acquired by measuring biomarkers in saliva. The use of the scanner, as reported in the literature, has limitations related to the operator’s experience and manual skills, the presence of blood and/or saliva during the impression, rescanning, and post-processing scans [92], and the accuracy scanner [67]. Meanwhile, the dosage of the biomarkers in the saliva cannot be influenced by the operator and is easy to measure. Therefore, biomarker monitoring could be useful in providing information relative to the bone remodeling processes during orthodontic treatment, even in the absence of evident clinical signs of tooth movement (assessed via clinical evaluation and/or through a scanner). This can be used to optimize orthodontic treatment in the individual subject with the choice of the most suitable forces to reduce treatment time [93,94,95,96].

## 5. Conclusions

The data obtained on the relationship between clinical and physiological factors that contribute to the changes and variability of salivary BTM concentrations after OTM stimulation are promising. In the first months of monitoring, the variations in the concentration of TRAcP and PINP on the salivary matrix in the individual subject, when compared with the RCV, provide information on the progress of the orthodontic treatment, especially in the absence of evident clinical signs. The use of BTMs on saliva could lead to the determination of the correct mechanical load in the OTM, improving durability and preventing adverse effects of the treatment.

## Figures and Tables

**Table 1:** Descriptive statistics of P1NP, PTHrP, and TRAcP concentrations in subjects evaluated. CI = Confidence Interval.

	P1NP (µg/L)	PTHrP (ng/mL)	TRAcP (U/L)
Number values	75	75	75
Mean	3.49	1.07	3.03
95% CI	3.10–3.87	0.90–1.24	2.69–3.38
Median	3.05	0.83	2.90
95% CI	2.75–3.50	0.68–0.91	2.40–3.40
Minimum	0.80	0.21	0.90
Maximum	8.84	2.94	6.93
D’Agostino–Pearson test for Normal distribution	reject Normality (*p* = 0.0019)	reject Normality (*p* = 0.0028)	reject Normality (*p* = 0.0092)

**Table 2:** Statistical evaluation of PINP, PTHrP, and TRAcP of time-stratified (T1, T2, and T3) concentrations. CI = Confidence Interval.

	PINP (µg/L)			PTHrP (ng/mL)			TRAcP (U/L)		
	T1	T2	T3	T1	T2	T3	T1	T2	T3
Number	25	25	25	25	25	25	25	25	25
Mean	2.13	3.13	5.22	0.57	1.04	1.61	1.76	3.30	4.05
95% CI	1.86–2.38	2.705–3.55	4.66–5.78	0.46–0.67	0.78–1.30	1.29–1.93	1.41–2.10	2.78–3.81	3.52–4.58
Median	2.10	2.90	5.20	0.55	0.83	1.57	1.50	3.29	3.90
95% CI	1.85–2.10	2.60–3.24	4.81–5.33	0.42–0.59	0.56–1.21	0.92–2.24	1.21–2.06	2.55–3.84	3.41–4.30
Minimum	0.80	1.54	2.90	0.21	0.34	0.52	0.90	1.20	2.01
Maximum	3.78	6.81	8.84	1.39	2.46	2.94	4.52	6.51	6.93
D’Agostino–Pearson test for Normal distribution	reject Normality (*p* = 0.1474)	accept Normality (*p* ≤ 0.0001)	reject Normality (*p* = 0.0537)	accept Normality (*p* = 0.0005)	reject Normality (*p* = 0.1027)	accept Normality (*p* = 0.016)	accept Normality (*p* = 0.0004)	reject Normality (*p* = 0.2756)	reject Normality (*p* = 0.4642)

**Table 3:** Mann–Whitney U test, used to compare the significance of the difference between the medians of the salivary biomarkers of subjects stratified by sampling period (T1, T2, and T3).

Variable	Mann–Whitney U Two-Tailed Probability
PINP T1 vs. PINP T2	*p* < 0.0001
PINP T2 vs. PINP T3	*p* < 0.0001
PINP T1 vs. PINP T3	*p* < 0.0001
PTHrP T1 vs. PTHrP T2	*p* = 0.0039
PTHrP T2 vs. PTHrP T3	*p* = 0.0091
PTHrP T1 vs. PTHrP T3	*p* < 0.0001
TRAcP T1 vs. TRAcP T2	*p* < 0.0001
TRAcP T2 vs. TRAcP T3	*p* = 0.0416
TRAcP T1 vs. TRAcP T3	*p* < 0.0001

**Table 4:** For each subject (a–ac), the percentage change in the concentration of the biomarkers measured at T1, T2, and T3 is reported. Non-significant percentage changes have been highlighted in light blue when compared with the specific RCV (21.18%, 68.44% 56.81%), for the analyte respectively for P1NP, PTHrP and TRAcP.

Subjects	P1NP Variation %			PTHrP Variation %			TRAcP Variation %		
	T1 vs. T2	T2 vs. T3	T1 vs. T3	T1 vs. T2	T2 vs. T3	T1 vs. T3	T1 vs. T2	T2 vs. T3	T1 vs. T3
a	13.38	73.11	96.28	125.00	17.68	164.77	123.08	20.69	169.23
b	53.19	81.60	178.19	182.76	15.45	226.44	45.30	46.19	112.42
c	36.05	64.10	123.26	69.78	24.58	111.51	125.54	15.55	160.61
d	10.71	67.74	85.71	81.82	101.67	266.67	184.17	24.93	255.00
e	92.50	116.88	317.50	61.90	126.47	266.67	91.33	6.62	104.00
f	118.75	51.43	231.25	68.97	38.78	134.48	100.00	21.25	142.50
g	38.10	79.31	147.62	29.27	73.58	124.39	45.22	15.87	68.26
h	14.29	216.67	261.90	6.12	201.92	220.41	74.44	55.41	171.11
i	40.00	97.32	176.25	35.96	97.52	168.54	55.36	13.56	76.43
l	53.85	50.00	130.77	61.54	50.00	142.31	203.08	5.33	219.23
m	61.71	130.39	272.57	118.42	62.65	255.26	119.33	20.97	165.33
n	53.85	61.25	148.08	25.42	41.89	77.97	76.09	14.07	100.87
o	14.29	20.83	38.10	201.92	4.46	215.38	100.00	11.67	123.33
p	23.81	107.69	157.14	130.56	12.05	158.33	116.67	8.97	136.11
q	118.75	37.14	200.00	68.97	6.12	79.31	75.00	38.10	141.67
r	36.84	100.77	174.74	36.36	86.67	154.55	132.00	11.21	158.00
s	27.17	109.83	166.85	85.92	90.15	253.52	101.89	13.32	128.79
t	33.33	91.67	155.56	244.64	16.58	301.79	17.93	6.45	58.00
u	13.64	64.31	86.71	51.72	201.14	356.90	38.89	56.00	116.67
v	87.50	60.00	200.00	5.13	56.10	74.10	33.33	308.33	444.44
z	139.79	13.95	173.24	15.56	75.00	102.22	66.19	23.50	105.24
y	3.44	40.15	44.97	4.35	27.78	33.33	105.56	15.68	137.78
w	114.29	24.89	167.62	52.54	106.67	215.25	141.67	17.24	183.33
ab	57.14	49.39	134.76	223.64	23.03	298.18	152.31	16.77	194.62
ac	38.10	38.10	71.43	36.36	137.33	223.64	48.15	13.75	68.52

## Data Availability

The data presented in this study are available from the corresponding author upon reasonable request.
